# Table olive yield and quality in super-high-density hedgerows: a long-term study

**DOI:** 10.3389/fpls.2026.1895003

**Published:** 2026-07-31

**Authors:** Ana Morales-Sillero, Pilar Rallo, María Rocío Jiménez, María Paz Suárez, Laura Casanova

**Affiliations:** Departamento de Agronomía, Escuela Técnica Superior de Ingeniería Agronómica (ETSIA), Universidad de Sevilla, Sevilla, Spain

**Keywords:** *Olea europaea* L., ‘Manzanilla de Sevilla’, ‘Manzanilla Cacereña’, hedgerows, yield efficiency, over-the-row harvester, fruit bruising

## Abstract

**Introduction:**

Olive cultivation in super-high-density orchards has expanded worldwide in olive groves for oil production, reducing cultivation costs and facilitating integral harvesting. Commercial expansion for table olive production is much more recent, but receives special attention given the increasingly scarce labor force. This study presents, for the first time, a long-term evaluation of olive production and quality in super-high-density hedgerows (3.75 m x 1.35 m) of two traditional cultivars, ‘Manzanilla de Sevilla’ and ‘Manzanilla Cacereña’.

**Methods:**

The hedgerows were grown under irrigation and identical environmental Mediterranean conditions; they were monitored for nine years from the fifth year after planting (2012-2020). Specifically, for each cultivar and year, olive yield, fruit quality, mechanical harvesting efficiency and dimensions of the hedgerows were studied. Besides, the growing habits of both cultivars were analyzed over three years and compared to those of adjacent ‘Arbequina’ hedgerows grown under similar conditions. This is a low vigor cultivar typically grown for oil production.

**Results/discussion:**

The results demonstrate that both the Manzanilla de Sevilla and Manzanilla Cacereña cultivars formed continuous and more vigorous than ‘Arbequina’. ‘Manzanilla Cacereña’ experiences a more restricted growth. This results in a narrower and shorter canopy that is significantly more productive and efficient, achieving a mean value of 15 t ha^-1^ and a cumulative yield of 136 t ha^-1^, 65% higher than that of ‘Manzanilla de Sevilla’ (89 t ha^-1^ cumulative yield; 10 t ha^-1^ mean). The efficiency of fruit removal exceeds 96% for both cultivars, but while ‘Manzanilla de Sevilla’ produces larger fruit with greater commercial value, it is extremely susceptible to mechanical damage—specifically browning and cuts—during harvesting. In contrast, ‘Manzanilla Cacereña’ stands out for its physical integrity, maintaining high fruit quality after mechanized harvesting.

## 1 Introduction

Global table olive production has shown a clear upward trend since 1990, tripling to exceed 3.3 million tons (IOC, 2026). Spain, Egypt, and Turkey are the leading producing countries, followed by others, mostly in the Mediterranean Basin. Besides, sustained growth has been observed in Asia, the Americas, and Africa. This trend parallels consumption over the same period, reaching 3 million tons (IOC, 2026), driven primarily by a perception of being a healthy food included in the Mediterranean diet. Table olives stand out for their nutritional composition, with high content in fiber and minerals such as iron, calcium, copper, and magnesium, and low sugars. In addition, they have a high oil content, considered a healthy fat rich in oleic acid, as well as other compounds with antioxidant capacity, as phenols and tocopherols, hydroxytyrosol, and vitamin E, among others (Rallo et al., 2018).

Olive cultivation is currently facing significant challenges, including the threat of low profitability in rainfed plantations, increased competition in the international market, and reduced competitiveness due to high production costs. In Spain, the world’s leading exporter of table olives and oil, the olive industry in general, and the table olive sector in particular, suffered in recent years a serious and structural labor shortage (ASEMESA, 2025). This notably affects olive harvesting and the performance of specialized tasks (such as pruning), leading to economic losses for farmers, increased production costs, and potential price increases for consumers. Therefore, the production of table olives in super-high-density hedgerows generates a growing interest due to the advantages it offers for mechanization.

Super-high-density cultivation of olive trees began in Spain in the 1990s for oil production and has now become the most widespread system in new and replanted orchards (also in other countries) (Díez et al., 2016; Gómez-del-Campo et al., 2022). Trees are usually planted at densities of 1,500 to 2,000 trees per hectare, with 3.5–4 m distance between rows and 1-1.5 m between trees in continuous rows, forming hedgerows that facilitate harvesting with over-the-row harvesters. This growing system has become increasingly prevalent in new olive groves as it responds to the need to reduce production costs (Penco-Valenzuela, 2020), increases labor efficiency, and admits full mechanization. The main drawbacks are the high investment required for the large number of trees per hectare, the installation of drip irrigation systems, and the need for more specialized technical management. The selection of early-bearing low-vigor cultivars, the appropriate planting density, water availability, and the management of pruning and harvesting, among others, are crucial to the profitability and sustainability of this type of grove (Camposeo et al., 2021; Vivaldi et al., 2015; García et al., 2017; Fernández et al., 2020; Cinosi et al., 2025; Trentacoste et al., 2021). Maintaining a well-balanced canopy structure is essential to improve light distribution and sustain high yield and fruit quality. The canopy architecture changes progressively as tree ages. It becomes larger and denser, therefore the light interception increases, but canopy porosity decreases and self-shading can become more pronounced reducing light availability in the inner and lower parts of the hedge. These changes can limit the renewal of productive shoots and reduce the photosynthetic activity of shaded leaves, which can lead to a gradual decline in productivity over time and may negatively affect fruit development, oil accumulation and fruit quality (Connor et al., 2014; Rosati et al., 2021; Trentacoste et al., 2021). To date, several studies focusing on the medium and long-term evolution of the performance of various cultivars in different environmental conditions, orchard designs, and agronomic management confirm the viability of this growing system intended for oil production (at least 10–14 years after planting) (Farinelli and Tombesi, 2015; Marino et al., 2017; Centeno et al., 2019; Díez et al., 2016; Gómez-del-Campo et al., 2017, 2020, 2022; Dias et al., 2022; Pérez-Rodríguez et al., 2024). Studies on hedgerow architecture have also shown the desirable characteristics of a cultivar for super-high-density cultivation. ‘Arbequina’ and ‘Arbosana’ (traditional Spanish cultivars widely used for this growing system) stand out among other cultivars by the lower vigor, smaller diameters of trunk, and higher branching frequency, exhibiting significantly finer lateral branches and annual shoots. This results in greater fruiting sites per unit of canopy volume (Rosati et al., 2013).

Table olive production has specific quality requirements. Olive fruit size, uniformity, pulp-to-pit ratio, firmness, and absence of damage (bruising) are among the factors that influence cultivar selection and agronomic management of commercial groves (Rallo et al., 2018). Traditionally, table olives were picked by hand to avoid bruising, as critical visual defects in the fruit cause commercial rejection. More recently, orchards were adapted to trunk vibrators to reduce cultivation costs, with the need of introducing specific postharvest treatments, such as a fast immersion in a cold diluted NaOH solution, to minimize the progression of fruit damage (Rejano et al., 2008; Zipori et al., 2021). The production of table olives in super-high-density conditions is now a commercial reality that has expanded in recent years due to the industry’s need to also reduce harvesting costs and address the growing labor shortage in order to avoid losing competitiveness. In this sense, a preliminary study conducted by the University of Seville in 2012 showed, for the first time, the adaptation of the traditional cultivars Manzanilla de Sevilla and Manzanilla Cacereña to this cultivation system (Morales-Sillero et al., 2014). Both Spanish cultivars are commonly used for table olive production, and their oils are also recognized for their quality (Morales-Sillero and García, 2015). The Manzanilla de Sevilla cultivar originated from Seville (Andalusia) and it is one of the most internationally recognized varieties of table olives for its high-quality fruit and good productivity. However, this contrasts with its high susceptibility to mechanical damage. ‘Manzanilla Cacereña’ is currently cultivated in northern Cáceres (Extremadura) and, despite being less well-known outside Spain, it is highly valued for both its fruit hardness and ease of harvesting (Barranco et al., 2005). The work mentioned (Morales-Sillero et al., 2014) was the starting point for commercial groves. Subsequent studies conducted on the same hedgerows focused on optimizing mechanical harvesting for table olive production (Jiménez et al., 2017; Pérez-Ruiz et al., 2018; Morales-Sillero et al., 2023) and evaluating fruit quality distribution on the hedgerows (Rallo et al., 2024) to improve the design and agronomic management. In recent years, the response of ‘Manzanilla de Sevilla’ hedgerows to different irrigation levels has been analyzed to demonstrate the cultivar suitability under deficit irrigation conditions (Morales-Sillero et al., 2024; Sánchez-Piñero et al., 2024).

Medium and long-term studies on the performance of the few table olive cultivars that are currently grown using super-high-density conditions are essential to meet the demands of growers and the industry. This is particularly important because, to date, no new table cultivars specifically selected for this growing system have been released from breeding programs (Rallo et al., 2026). In this regard, the main objective of this work is to analyze the evolution of the groves of Manzanilla de Sevilla and Manzanilla Cacereña cultivars from the fifth year after planting and over a period of nine years, with particular attention to fruit yield, fruit quality before and after mechanical harvesting, fruit removal efficiency, and hedgerow development. In addition, the architecture of the hedges is analyzed in comparison with that of ‘Arbequina’.

## 2 Materials and methods

### 2.1 Experimental site

**Figure 1:**
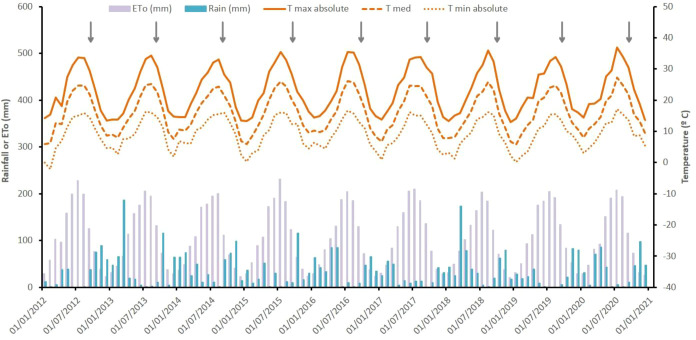
Average values of medium, maximum and minimum temperatures, monthly rainfall and reference evapotranspiration (ET_0_), from January 2012 to December 2020. ↓, Harvesting date.

The study was carried out from 2012 to 2020 in an irrigated orchard located in Portugal (Campo Maior; latitude: 38° 55 ′ 55.1 ′ N; longitude: 7° 02 ′ 36.8 ′ W; altitude: 201 m (WGS84). The cultivars were planted in 2007 in adjacent plots, each covering approximately 1 ha. Hedgerows were oriented in a North-South direction, with a tree density of 1,975 trees ha^-1^ and a spacing of 3.75 m x 1.35 m. The climate is Mediterranean. The mean values of monthly reference evapotranspiration (ET_0_), rainfall, and temperature (**Figure 1**) were registered for the same period in a nearby weather station (latitude: 38° 52 ′ 37 ´´ N; longitude: 6° 49 ´40 ´´ W; altitude: 188 m). Mean annual were 1,278.7 mm and 421.2 mm, respectively. The rainfall varied between 265.3 mm in 2017 and 571.9 mm in 2018. The average annual, absolute maximum and minimum temperature were 15.7, 23 and 9 °C, with up to 42.9 °C in August 2018 and -6.1 °C in February 2012. Soil texture was a loam, with pH 7.4. Until 2015, the effective soil depth of the grove was 0.6 m, and the irrigation system consisted of one drip line per tree row with two 2.43 L h^–1^ compensating drippers per tree, separated 0.60 m. In 2016, raised beds ≈0.6 m high were built in the tree rows to increase soil depth and two drip lines with 1.2 L h^–1^ drippers, spaced 0.5 m apart, were installed.

A standard commercial practice was applied for the management of the hedgerows. The amount of water applied was around 300 mm, with a minimum of 238 mm in 2019 and a maximum of 364 mm in 2018. The irrigation period usually began in March and ended in October. Fertigation was guided by previous foliar analyses to non-limiting nutrient conditions. Soil management consisted in natural groundcover between tree rows and the application of non-residual herbicides to control weeds in the tree rows. The trees were trained to a central leader system, and pruning was performed each year in winter to keep the dimensions of hedgerows suitable for mechanical harvesting. Until 2014, pruning consisted in the manual removal of the branches growing toward the center of the rows and mechanical topping to 2.6 m height. The lower branches of the trees were pruned to 0.6 m above ground using mechanical hedge trimmers. From 2015, mechanical pruning was made on one side of the hedges every two years with angled discs to achieve greater radiation interception. Manual pruning was carried out after mechanical pruning to remove old, diseased, or poorly positioned branches. The pruning debris was shredded and spread over the center of the tree rows.

Each year, three rows per cultivar consisting of 90–150 trees were randomly selected for fruit yield, fruit quality before and after mechanical harvesting, and hedgerows dimensions analysis. They were considered experimental units.

Fruits were harvested once the skin color turned green-yellowish, that is at maturity index 1 (Ferreira, 1979), which is considered optimum for Spanish-style green olive elaboration. This occurred usually in the second half of September, except in 2018 when the harvest of both cultivars had to be postponed until October 9 due to the rains in the previous weeks. For each experimental unit, a ≈3 kg sample was randomly hand-picked for fruit quality assessment (Hand harvesting sample). The remaining fruits were then harvested using an over-the-row harvester (New Holland Braud 9090X Olive, CNH Global, Zedelgem, Belgium). Prior to unloading the olives from the harvester into a trailer, a 20 kg subsample, consisting of approximately equal amounts of fruit from each of the two hoppers, was collected. Subsequently, a 3 kg sample was taken from this subsample for fruit quality analysis (Mechanical harvesting sample). The nominal speed and the beating frequency of the harvester were 3.5 km h^-1^ and 480 r.p.m., respectively, in the 2012–2014 period, and 2 km h^-1^ and 430 r.p.m from 2015 to 2020, in agreement to previous research. Foliar applications of cooper-based fungicide were made immediately after mechanical harvest and pruning. At the beginning of the studied period, a high incidence of *Pseudomonas savastanoi* was observed in the hedgerows of both cultivars.

### 2.2 Production and fruit characteristics

Mean fruit yield (t ha^-1^) per experimental unit was estimated according to tree density and from the total weight of the fruits, considering the fruits removed by the harvester, the fruits that remained on trees, and, from 2016 to 2020, the fruits that fell on ground. For the year 2013, only production data is available. This data was provided for each cultivar and plot by the company that owns the plantation (no statistical analysis could be performed).

The index of alternate bearing (ABI) for fruit yield was calculated for the period 2014-2020 to analyze the influence of cultivar following Pearce and Dobersek-Urbanc (1967).


$$ABI=\frac{1}{n-1}\sum_{n=1}^{n}\left|\frac{a_{n+1}-a_{n}}{a_{n+1}+a_{n}}\right|$$

Where n is the number of the studied years (7) and a_i_ is the yield for year n.

Mean fruit weight (g) was measured from 0.5 kg fruit subsamples per experimental unit. Other fruit traits were measured using the same samples: fruit volume (mL) was determined from the volume displaced after immersion of the samples in graduated cylinders filled with water, and pulp-to-pit ratio in fresh weight was estimated from the difference between the weights of fruit (g) and pits (g). Besides, fruit shape and symmetry were determined in samples of 50 fruits, the first one as the ratio between the maximum longitudinal and equatorial diameters (mm), and the second one by classification in three categories: symmetrical, slightly symmetrical and asymmetrical. Color index was determined in 30 fruit subsamples (Castellano et al., 1993). Measurements on the equatorial zone of each fruit were made using a Minolta CM-700d (Konica Minolta Inc., Tokyo, Japan) spectrophotometer. The International Commission on Illumination color notation system was applied to determine the L* (lightness), a* (color axis from green to red) and b* (color axis from blue to yellow).

The proportion of fruits with cuts (%), non-bruised fruits (%) and bruising incidence (BI) were estimated in fruit subsamples of 100 fruits two hours after manual and mechanical harvesting. The BI was calculated from non-bruised fruits (No), low damaged fruits (< 25% of the skin surface, Nl) and severely damaged fruits (≥25% of the skin affected, Ns) (Morales-Sillero et al., 2014), as follows:


$$BI=\frac{Nl+Ns}{No+Nl+Ns}$$

Fruit size distribution was obtained, from 2014 to 2020, from the unitary weight (g) in a subsample of 100 fruits. Six classes based on fruit number per kilo were established: Extra Large (<200); Large (220–240); Medium and Small (241–300); Petit (301–400); Subpetite (401–420) and smaller than subpetite (>420), the latter considered commercially unsuitable by the table olive industry.

### 2.3 Harvesting efficiency

Fruit removal (%) per experimental unit was estimated from the total yield, the weight of fruits that remained after mechanical harvest on 10–12 random trees (depending on year), and, from 2016 to 2020, from the proportion of fruits that fell on ground, estimated from the weight of fruits that dropped on a specific surface (2.5–5 m x 2.5 m, also depending on year). The average harvest time (expressed in h ha^–1^) was determined measuring the time needed by the harvester to complete each row of trees.

### 2.4 Hedgerows dimensions and architectural traits

Tree height (m), plant width at 0.80 m and 1.70 m above ground, and trunk diameter (mm) at 0.30 m, were determined the day before harvesting in 10 randomly selected trees per experimental unit. Each year, External Surface Area (ESA) and canopy volume were obtained considering a rectangular prism shape. Hedgerow porosity (%) was estimated by image analysis of digital pictures obtained with a Nikon D600 reflex camera (Nikon Corp., Tokyo, Japan) and processed with a CobCal software ver. 2.0 (Bs As, Argentina). A red sheet was previously placed in the background of the hedgerow.

For each experimental unit, the fruit number was estimated from fruit yield and fruit weight. Subsequently, yield and fruit efficiency were estimated per trunk diameter, external surface area and canopy volume.

Architectural traits were also measured in five randomly selected trees per cultivar on 25/01/2017, 08/01/2018, and 28/01/2019, before pruning. The number of branches growing from the central leader and their basal diameters (cm) (two perpendicular measurements) were taken at heights between 0.90 m and 1.5 m. Subsequently, the number of current shoots on each of the three basal branches in this window was counted, and, on four of these shoots, the length, number of nodes, and basal diameter were measured. All these measurements were also taken on the same number of ‘Arbequina’ trees that were planted in 2007 in adjacent hedgerows, with the same pattern and managed under similar conditions.

### 2.5 Statistical analysis

Each year, one-way ANOVA test was performed for all parameters to compare between the cultivars. Additionally, a multivariate analysis was conducted including the year as a factor to study its effect (environmental conditions) and the interaction (cultivar x year). To achieve variance normality and homogeneity, data were previously transformed using the Box-Cox power transformations when necessary (Box and Cox, 1964). The percentages were normalized using the arcsine square root transformation. Tukey’s Test (P<0.05) was used to discriminate mean values when significant differences were found. Mean values of bruising incidence were calculated and contingency tables produced. Linear regressions were studied between different parameters as well as the coefficient of determination (R^2^). StatGraphics 19 software was used for all statistical analysis.

## 3 Results

### 3.1 Fruit quality

A comparative analysis revealed significant differences between cultivars in most years of the period studied (2012-2020) and that the interaction between both factors, the cultivar (genetic) and year (the environmental factor), is determinant in fruit quality (**Table 1**). In terms of mean values, the ‘Manzanilla de Sevilla’ fruits had higher weight (mean values of 4.0 g, ranged between 3.6 g in 2012 and 4.6 g in 2016) and volume (4.0 mL, ranged between 3.8 mL in 2012, 2015, 2017 and 2019 and 4.6 mL in 2016) compared to the ‘Manzanilla Cacereña’ fruits (mean value of 3.7 g of fruit weight, ranged between 2.9 g in 2012 and 4.4 g in 2019; 3.6 mL of mean fruit volume, ranged between 3.1 mL and 4.3 mL in the same years). The mean values of pulp to pit ratio were 7, and this parameter varied from 5.8 in 2012 to 8.4 in 2018 in ‘Manzanilla de Sevilla’ fruits. ‘Manzanilla Cacereña’ on the other hand, ranged between 6.0 in 2014 and 7.5 in 2019.

**Table 1:** Fruit traits for the studied cultivars growing in super-high-density hedgerows from the fifth to the 13^th^ year after plantation.

Cultivar	Year	Fruit weight (g)	Fruit volume (mL)	Pulp-to-pit ratio	Shape index	Fruit simmetry	Color index	Hand harvesting	
								Non-bruised fruit (%)	Bruising incidence
‘Manzanilla de Sevilla’	2012	3.6 b	3.8 b	5.8	1.19	1.4 a	25.8	51.0 a	0.51 b
	2014	3.9 b	4.0 b	6.1				91.3 a	0.09
	2015	3.8	3.8	6.8	1.20	1.7 a	31.0 b	77.3 a	0.23 b
	2016	4.6	4.6	7.3	1.18	1.5	28.8 b	78.7	0.21
	2017	3.8	3.8	7.6	1.22 a	1.8 a	30.5 b	87.0	0.13
	2018	4.4 b	4.4 b	8.4 b	1.19 a	2.2	31.3 b	83.9	0.17
	2019	3.8 a	3.8	6.5 a	1.25	1.7	27.4	79.5 a	0.21 b
	2020	4.1 b	4.1 b	7.0	1.24	1.8	26.3 b	92.8	0.07
	Mean	4.0	4.0	7.0	1.21	1.7	28.8	80.2	0.22
‘Manzanilla Cacereña’	2012	2.9 a	3.1 a	6.2	1.20	1.8 b	25.6	91.3 b	0.09 a
	2014	3.3 a	3.3 a	6.0				96.7 b	0.03
	2015	3.7	3.6	6.7	1.17	2.0 b	29.3 a	92.0 b	0.08 a
	2016	4.3	4.3	7.2	1.22	2.1	24.5 a	83.7	0.17
	2017	3.7	3.6	7.4	1.27 b	2.6 b	27.3 a	90.3	0.10
	2018	3.5 a	3.5 a	7.0 a	1.29 b	2.3	27.8 a	92.7	0.10
	2019	4.4 b	4.3	7.5 b	1.28	1.8	27.7	92.3 b	0.08 a
	2020	3.6 a	3.5 a	7.2	1.23	1.7	25.1 a	94.8	0.05
	Mean	3.7	3.6	6.9	1.24	2.0	26.8	91.7	0.09
Cultivar		****	****	ns	****	****	****	****	****
Year		****	****	****	****	****	****	****	****
Cultivar x Year		****	***	***	****	***	***	****	****

Fruit symmetry refers to the similarity between the two halves separated by the vertical axis of the fruit (1: Symmetrical fruit; 2: Slightly asymmetrical fruit; 3: Asymmetrical fruit). Different lowercase letters indicate significant differences between cultivars for each year by Tukey test (P<0.05) in one-way ANOVA test. The significance of the last three lines corresponds to the multivariate analysis: ns, non-significant differences. ***, **** significant differences at P< 0.001 and 0.0001, respectively. Fruit shape is measured as the ratio between fruit length and width.

Concerning appearance, ‘Manzanilla de Sevilla’ fruits were considerably the most symmetrical (mean index value of 1.7 compared to 2.0 in ‘Manzanilla Cacereña’) and slightly less elongated, as indicated by the mean shape index values (1.21 and 1.24, respectively). The color index was markedly higher in ‘Manzanilla de Sevilla’ (28.8) than in ‘Manzanilla Cacereña’ (26.8). In ‘Manzanilla de Sevilla’ the lowest value was found in 2012 (25.8) and the greatest in 2018 (31.3). In ‘Manzanilla Cacereña’ the lowest value was found in 2016 (24.5) and the greatest in 2015 (29.3). After manual harvesting, significant differences between cultivars were also noticed. In this case, the Manzanilla Cacereña cultivar was notably more resistant to bruising, with 91.7% of undamaged fruits (83.7% in 2016-96.7% in 2014) and a bruising incidence (BI) of 0.09 (0.03 in 2014-0.17 in 2016), compared to 80.2% of undamaged fruits in the Manzanilla Sevilla cultivar (51% in 2012-92.8% in 2020) and 0.22 of BI (0.07 in 2020-0.51 in 2012).

**Figure 2:**
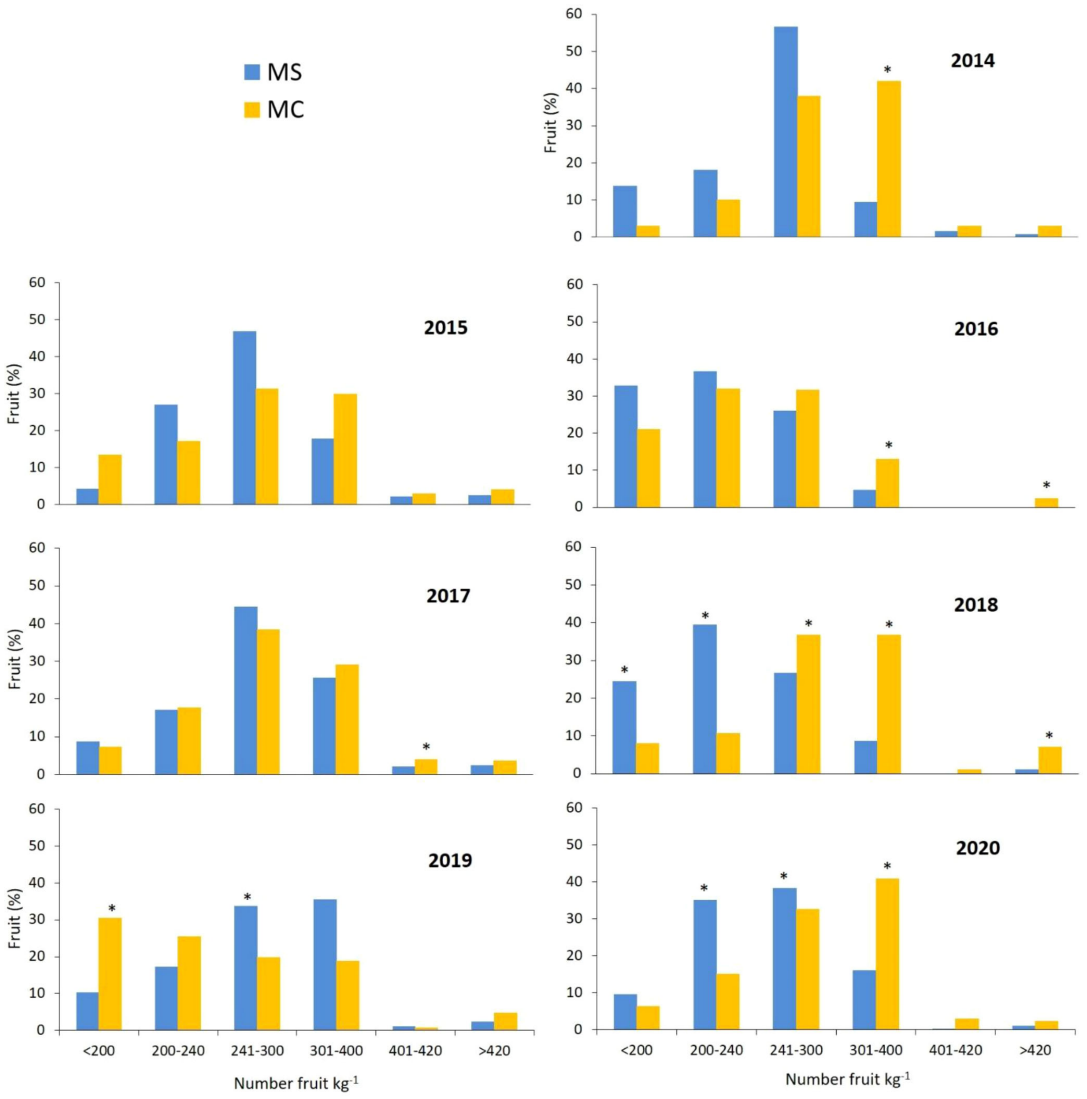
Mean values of fruit size distribution of ‘Manzanilla de Sevilla’ and ‘Manzanilla Cacereña’ high-density hedgerows. The number of fruits per kg were classified based on five classes: Extra-Large (<200) Large (220–240); Medium and Small (241–300); Petit (301–400); Subpetit (401–420) and smaller than subpetite (*>*420). *significant differences between varieties (Tukey test *P* < 0.05).

The distribution of commercial sizes (number of fruits per kilogram) varied considerably between cultivars and years, reflecting a trend for ‘Manzanilla de Sevilla’ towards larger sizes (fewer fruits kg^-1^) and for ‘Manzanilla Cacereña’ towards medium and small sizes (**Figure 2**). Thus, Manzanilla de Sevilla’ showed a higher percentage of fruits in the large size categories, specifically in the ranges of <200, 200-240 and 241–300 fruits per kilogram. The distribution of ‘Manzanilla Cacereña’ fruits showed the greatest interannual variability and was more shifted to the right, with a frequent predominance in the 241–300 and 301–400 fruit kg^-1^ ranges, except in 2016 and 2019. Differences between cultivars were important for certain sizes and years.

### 3.2 Production

**Figure 3:**
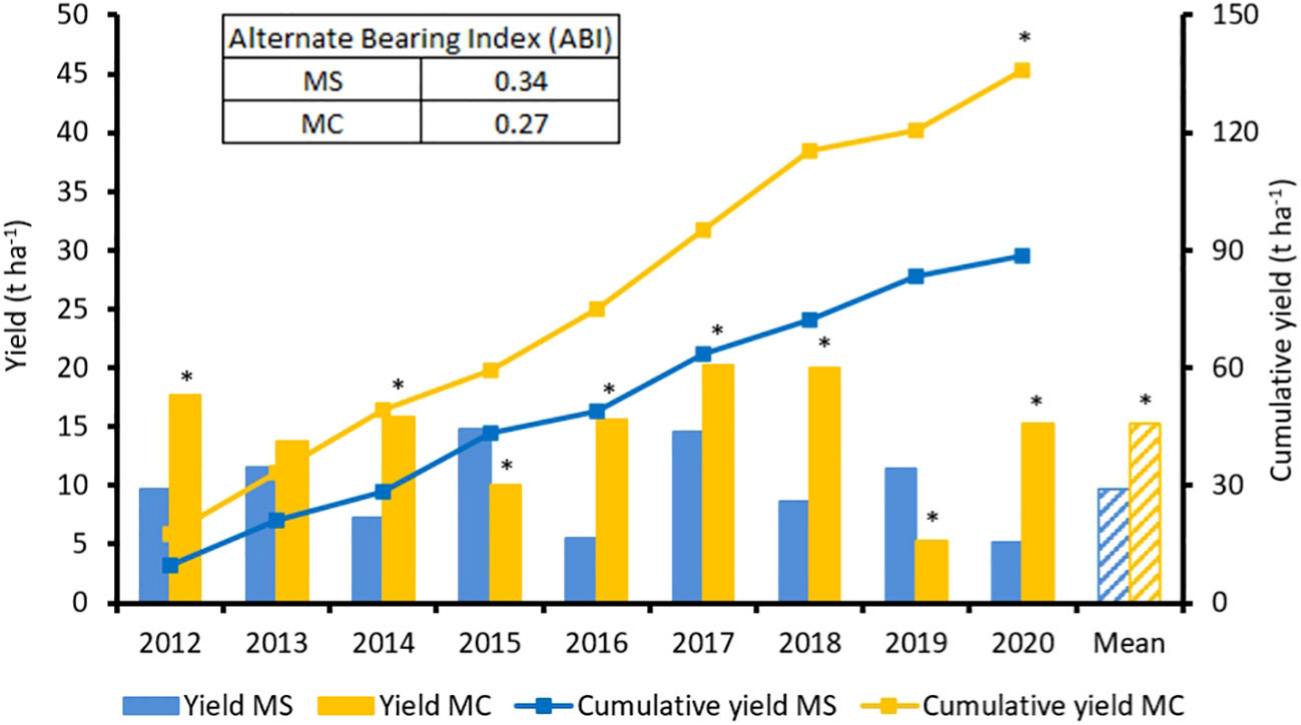
Mean values of fruit yield per year and the studied period, and cumulative yield (t ha^-1^) for ‘Manzanilla de Sevilla’ (MS) and ‘Manzanilla Cacereña’ (MC) in super-high-density hedgerows from 2012 to 2020. The orchards were planted in 2007 (Campo Maior, Portugal). *significant differences between cultivars (Tukey test *P* < 0.05); The 2013 average data for each plantation was provided by the company owning the field site (no statistical analysis was made). The Alternate Bearing Index (ABI) was calculated for the 2014–2020 period, and no significant differences between varieties were found.

Cultivar and year greatly influenced fruit yield in the 2012–2020 period studied. ‘Manzanilla Cacereña’ was the most productive with mean values of 7.6 kg tree ^-1^, considerably greater compared to ‘Manzanilla de Sevilla’ (5.0 kg tree^-1^). In terms of yield per hectare (**Figure 3**), ‘Manzanilla Cacereña’ hedgerows reached a mean value of 15 t ha^-1^, maximums of 20 t ha^-1^ in 2017 and 2018 and minimums usually equal to or greater than 10 t ha^-1^, except for 5 t ha^-1^ in 2019. ‘Manzanilla de Sevilla’ hedgerows had a mean value of 10 t ha^-1^, a maximum of 15 t ha^-1^ in 2015 and 2017 and a minimum of approximately 5.0 t ha^-1^ in 2020. Cumulative production at the end of the period was 136 t ha^-1^ in ‘Manzanilla Cacereña’, higher than the 89 t ha^-1^ of ‘Manzanilla de Sevilla’.

Production stability was estimated using the alternate bearing index (ABI). For this index, a low value is desirable. Greater yield irregularities were found in the hedges of ‘Manzanilla de Sevilla’, with and average ABI value of 0.34, compared to ‘Manzanilla Cacereña’ (0.27). This alternation or biennial bearing was apparently clear in the first cultivar but the differences between cultivars were not significant. Besides, fruit production cycles were not synchronized. ‘Manzanilla de Cacereña’ usually showed an opposite pattern of fruiting alternation.

**Figure 4:**
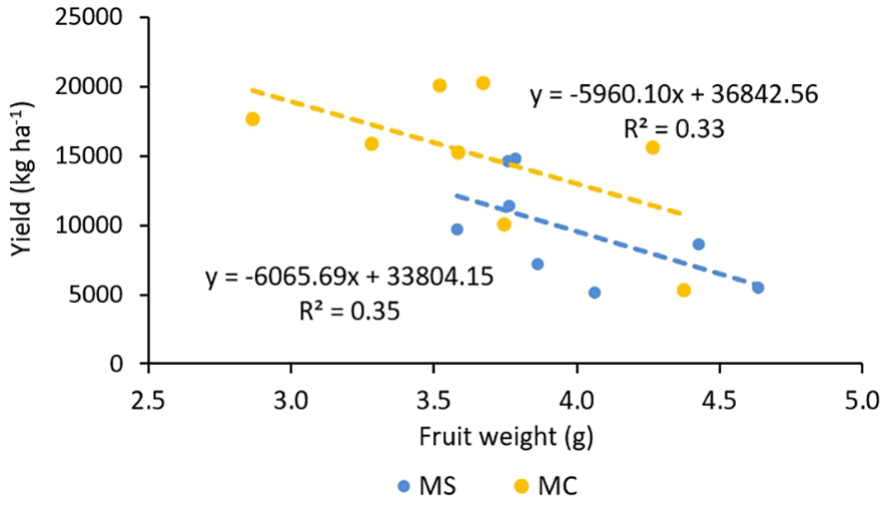
Relationship between fruit yield and fruit weight of ‘Manzanilla de Sevilla’ (MS) and ‘Manzanilla Cacereña’ (MC) in super-high-density hedgerows from 2012 to 2020 (except for 2013). The orchards were planted in 2007 in Campo Maior (Portugal).

The alternate fruit bearing played a fundamental role in the fruit size distribution (**Figure 2**): In low-production years, such as 2016 for ‘Manzanilla de Sevilla’ and 2019 for ‘Manzanilla Cacereña’, the percentage of fruit in the larger sizes (<200 mm and 201–240 mm) increased considerably. In high-yield years, such as 2018 for ‘Manzanilla Cacereña’, the fruit tended to shift towards smaller sizes (301–400 mm). A negative relationship between total yield and fruit weight was found for both cultivars (R*^2^* = 0.35 for ‘Manzanilla de Sevilla’ and 0.33 for ‘Manzanilla Cacereña’), suggesting that more abundant crops tend to produce smaller fruits (**Figure 4**).

### 3.3 Harvesting efficiency and mechanical damage of fruits

The mechanical harvesting performance was evaluated using key indicators such as the percentage of fruit removed by the over-the row harvester, the amount of fruit that fell to the ground, and the time spent per hectare. A fundamental aspect of the study was the physical integrity of the fruits, for which the bruising incidence (BI) and the percentage of fruit with cuts were determined. For all these parameters, significant differences were found between cultivars, and the interaction between cultivar and year was important only for the bruising measurement (**Table 2**).

**Table 2:** Harvesting efficiency and fruit damage after mechanical harvesting for the studied cultivars growing in super-high-density hedgerows from the fifth to the 13^th^ year after plantation.

Cultivar	Year	Fruit removal (%)	Fruit on ground (%)	Time to harvest (h ha^-1^)	Mechanical harvesting		
					Non-bruised fruits (%)	Bruising incidence	Cut fruits (%)
‘Manzanilla de Sevilla’	2012	97.7		1.1	0.0 a	1.9 b	17.7 b
	2014	97.4		1.8	0.0 a	1.6 b	10.5 b
	2015	99.6		1.5	0.3 a	1.6 b	7.0
	2016	98.8	3.3	1.5 a	0.0	1.6 b	9.7 b
	2017	98.4	1.9	1.8	0.7 a	1.7 b	5.3
	2018	98.2	2.2	1.7	0.8 a	1.5 b	12.3 b
	2019	97.1	2.6	2.0	0.3 a	1.9 b	17.8 b
	2020	99.4 b	2.9	1.5	0.0 a	1.9 b	11.5
	Mean	98.3	2.6	1.6	0.3	1.7	11.5
‘Manzanilla Cacereña’	2012	98.0		1.6	9.0 b	1.1 a	2.0 a
	2014	96.3		2.0	4.0 b	1.1 a	2.3 a
	2015	98.2		1.7	7.3 b	1.1 a	3.6
	2016	96.8	2.3	1.8 b	2.0	1.3 a	1.7 a
	2017	96.3	1.6	1.9	7.0 b	1.3 a	1.0
	2018	97.4	1.3	1.9	13.3 b	1.0 a	0.0 a
	2019	97.0	2.3	1.7	1.5 b	1.7 a	6.8 a
	2020	97.0 a	1.9	1.6	13.0 b	1.1 a	2.3
	Mean	97.1	1.9	1.8	7.1	1.2	2.5
Cultivar		*	*	**	****	****	****
Year		ns	ns	**	****	****	**
Cultivar x Year		ns	ns	ns	****	****	ns

Fruit on ground data were determined from 2016 onwards.

Different lowercase letters indicate significant differences between cultivars for each year by Tukey test (P<0.05) in one-way ANOVA test. The significance of the last three lines corresponds to the multivariate analysis: ns, non-significant differences. *, ** and ****. significant differences at P< 0.05, 0.01 and 0.0001, respectively.

**Figure 5:**
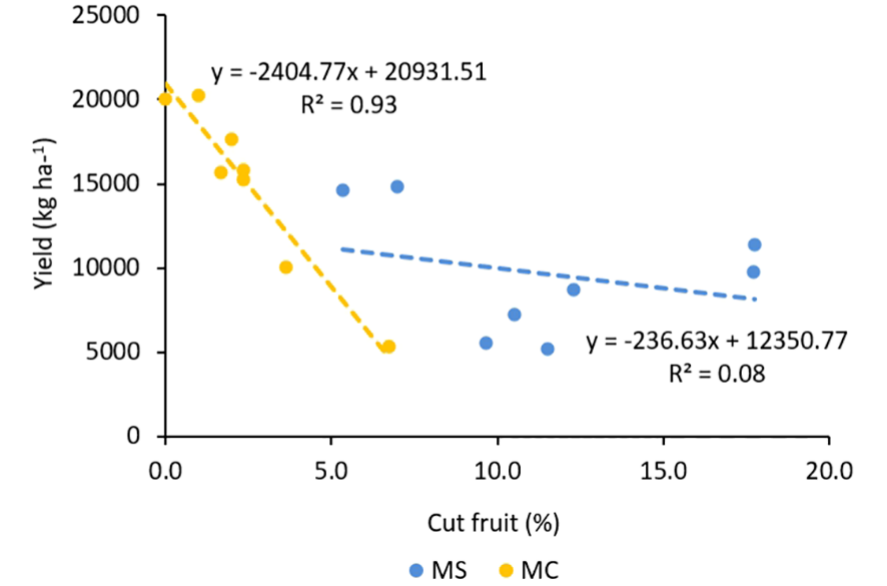
Relationship between fruit yield and the percentage of cut fruit of ‘Manzanilla de Sevilla’ (MS) and ‘Manzanilla Cacereña’ (MC) in super-high-density hedgerows from 2012 to 2020 (except for 2013). The orchards were planted in 2007 in Campo Maior (Portugal).

Fruit removal efficiency was always very high, although ‘Manzanilla de Sevilla’ had a slightly higher average of 98.3% (with a maximum of 99.6% in 2015), while ‘Manzanilla Cacereña’ reached 97.1% (maximum of 98.2% in 2015). The amount of fruit falling to the ground, that was estimated from 2016 onwards, was low in both cultivars and significantly higher for ‘Manzanilla de Sevilla’ (2.6%, ranged between 1.9 in 2017 and 3.3 in 2016) than for ‘Manzanilla Cacereña’ (1.9%, ranged between 1.3 in 2018 and 2.3 in 2016 and 2019). The time required to harvest a hectare of ‘Manzanilla de Sevilla’ was 1.6 h on average (1.1 h in 2012-2.0 h in 2019), compared to 1.8 h for ‘Manzanilla Cacereña’ (1.6 h in 2012 and 2020-2.0 h in 2014). After mechanical harvesting, the percentage of non-bruised ‘Manzanilla de Sevilla’ fruit was practically zero (up to 0.8% in 2018), while in ‘Manzanilla Cacereña’ it was 7.1% and ranged between 1.5% in 2019 and around 13% in 2018 and 2020. The BI had an average value of 1.7 for ‘Manzanilla de Sevilla’ and ranged between 1.5 in 2018 and 1.9 in 2012, 2019 and 2020. This index was always significantly higher than for ‘Manzanilla Cacereña’, which showed an average value of 1.2 (1.0 minimum in 2018 and 1.7 maximum in 2019). The percentage of fruits with cuts also differed considerably between cultivars in most years, with ‘Manzanilla de Sevilla’ showing the highest damage, with a mean value of 11.5% (ranged from 5.3% in 2017 to 18% in 2012 and 2019), compared to 2.5% recorded for ‘Manzanilla Cacereña’ (ranged from 0% in 2018 to 6.8% in 2019). A very strong negative correlation (R² = 0.93) was found between fruit yield and cut fruits only in this cultivar (**Figure 5**). In ‘Manzanilla de Sevilla’, despite the high incidence of cut fruits, an almost negligible correlation with fruit yield was obtained (R² = 0.08).

### 3.4 Hedgerows structure

Statistical analysis revealed important differences between cultivars based on hedge structure, and the interaction between cultivar and year for most of the parameters studied (**Table 3**). Continuous hedgerows were already evident five years after planting, at the beginning of the study. ‘Manzanilla de Sevilla’ hedgerows stood out for being more vigorous and showing higher canopy porosity, although differences between cultivars were not significant every year. ‘Manzanilla de Sevilla’ hedgerows had an average height of 2.7 m (2.5-2.8) and a width of 1.6 m (1.4-2.0). ‘Manzanilla Cacereña’ hedgerows reached an average height of 2.5 m (2.4-2.7 m) and a width of 1.4 m (1.3-1.6 m). The trunk diameter was also higher in ‘Manzanilla de Sevilla’ hedgerows (average value of 88.3 mm) and increased from 69.1 mm in 2012 to a maximum of 99.2 mm in 2019. For the ‘Manzanilla Cacereña’ hedges, the average value was 82.2 mm, and it increased from 66.5 mm in 2012 to a maximum of 93.3 mm in 2020.

**Table 3:** Hedgerows for the studied cultivars growing in super-high-density hedgerows from the fifth to the 13^th^ year after plantation.

Cultivar	Year	Tree height (m)	Hedgerow width (m)	Trunk diameter (mm)	External Surface Area		Canopy volume		Porosity (%)	Yield efficiency				Fruit efficiency	
					(m^2^ plant^-1^)	(m^2^ ha^-1^)	(m^3^ plant^-1^)	(m^3^ ha^-1^)		/Trunk (g mm^-1^)	/ESA (g m^-2^)	/Vol (g m^-3^)	/Trunk (No mm^-1^)	/ESA (No m^-2^)	/Vol (No m^-3^)
‘Manzanilla de Sevilla’	2012	2.8 b	2.0 b	69.1	9.2 b	18105.8 b	6.4 b	12618.9 b		63.7 a	480.5 a	689.5 a	17.6 a	132.4 a	190.0 a
	2014	2.6	1.6 b	87.5	8.7	17226.2	5.2 b	10360.5 b		41.2 a	416.5 a	686.6 a	10.6 a	106.3 a	175.1 a
	2015	2.8 b	1.4	86.3	8.8 b	17294.2 b	4.8 b	9464.2 b		86.7 b	854.6 b	1567.5	23.0 b	226.7 b	414.8 b
	2016	2.5	1.5	85.0	7.8	15379.8	4.1	8055.6	31.3	32.8 a	357.8 a	686.4 a	7.1 a	77.5 a	149.1 a
	2017	2.7	1.5	92.3 b	8.7	17195.1	4.9	9696.3	19.5 b	80.5 a	850.7 a	1512.6 a	21.5 a	226.5 a	402.8 a
	2018	2.7	1.5	88.9	8.8	17452.8	5.6	11129.3	21.8 b	49.4 a	497.0 a	783.3 a	11.2 a	112.3 a	177.0 a
	2019	2.7	1.7 b	99.2 b	8.9	17630.8	5.6	11000.9	32.4	58.3 b	647.6 b	1041.3 b	15.9 b	176.8 b	284.5 b
	2020	2.7 b	1.5	98.3	8.7	17092.9	4.9	9750.9	20.7 a	26.6 a	301.8 a	529.1 a	6.5 a	74.3 a	130.2 a
	Mean	2.7	1.6	88.3	8.7	17172.2	5.2	10259.6	25.1	54.9	550.8	937.0	14.2	141.6	240.5
‘Manzanilla Cacereña’	2012	2.4 a	1.5 a	66.5	7.5 a	14781.0 a	4.0 a	7875.8 a		150.9 b	1336.4 b	2540.2 b	53.0 b	469.0 b	890.2 b
	2014	2.6	1.4 a	86.1	8.3	16480.5	4.4 a	8723.2 a		96.7 b	997.6 b	1912.6 b	29.6 b	304.3 b	582.2 b
	2015	2.5 a	1.3	78.1	8.1 a	15938.0 a	4.1 a	8094.2 a		65.5 a	633.1 a	1249.7	17.5 a	169.0 a	333.0 a
	2016	2.4	1.4	83.0	7.7	15140.7	3.7	7365.4	30.7	97.8 b	1033.9 b	2125.4 b	22.8 b	241.5 b	496.5 b
	2017	2.7	1.4	78.2 a	8.6	17019.6	4.8	9527.5	12.3 a	131.3 b	1191.9 b	2132.5 b	35.9 b	324.9 b	581.0 b
	2018	2.7	1.5	86.2	8.6	16990.7	5.2	10273.9	12.4 a	118.1 b	1179.6 b	1961.0 b	33.6 b	335.4 b	556.0 b
	2019	2.7	1.6 a	86.4 a	8.8	17407.9	5.2	10204.1	28.2	31.9 a	312.5 a	534.4 a	7.1 a	69.5 a	118.8 a
	2020	2.6 a	1.4	93.3	8.7	17163.1	4.9	9624.2	31.7 b	82.7 b	888.0 b	1595.4 b	23.1 b	249.0 b	448.0 b
	Mean	2.5	1.4	82.2	8.3	16365.2	4.5	8961.0	23.1	96.9	946.6	1756.4	27.8	270.3	500.7
Cultivar		****	****	****	****	****	****	****	**	****	****	****	****	****	****
Year		****	****	****	****	****	****	****	****	****	****	****	****	****	****
Cultivar x Year		***	****	*	****	****	****	****	****	****	****	****	****	****	****

Porosity was determined from 2016 onwards. ESA: External Surface Area.

Different lowercase letters indicate significant differences between cultivars for each year by Tukey test (P<0.05) in one-way ANOVA test. The significance of the last three lines corresponds to the multivariate analysis: ns, non-significant differences. *, **, *** and ****, significant differences at P< 0.05, 0.01, 0.001 and 0.0001.

The differences in geometry explained the narrower canopy of ‘Manzanilla Cacereña’, with the mean values of ESA (External Surface Area) and canopy volume of 16365.2 m² ha^-1^ and 8961.0 m^3^ ha^-1^, respectively (**Table 3**) in contrast to the 17172.2 m² ha^-1^ and 10259.6 m^3^ ha^-1^ of ‘Manzanilla de Sevilla’. Concerning porosity, the mean value was significantly higher in ‘Manzanilla de Sevilla’ hedges, with 25.1% (19.5-32.4%) than in the ‘Manzanilla Cacereña’ (23.1%, 12.4-31.7%).

Yield Efficiency estimated in terms of trunk diameter, ESA and canopy volume, was greater for ‘Manzanilla Cacereña’ (**Table 3**), which produced 96.9 g mm^-1^ of trunk diameter, 946.6 g m^-2^ of ESA, and 1756.4 g m^-3^ of canopy volume, around 34%, 42% and 47%, respectively greater than in ‘Manzanilla de Sevilla’ (54.9 g mm^-1^, 550.8 g m^-2^, and 937.0 g m^-3^). In exceptional years, like 2012, the ‘Manzanilla Cacereña’ produced up to 2540.2 g m^-3^.

Efficiency was also estimated using the number of fruits per tree. Results show that Manzanilla Cacereña cultivar also exhibited a higher fruit density, with 27.8 fruits mm^-1^ of trunk diameter, 270.3 fruits m^-2^ of ESA, and 500.7 fruits m^-3^, compared to the 14.2 fruits mm^-1^, 141.6 fruits m^-2^, and 240.5 fruits m^-3^ of ‘Manzanilla de Sevilla’.

Architectural traits were measured in this study over three years (2017-2019) for both cultivars and for adjacent hedgerows of ‘Arbequina’ growing in similar conditions and with the same age, design pattern and management (**Table 4**). No differences were found in the average values between the two Manzanilla cultivars in any of the parameters studied, but mean values were substantially different to that of ‘Arbequina’. The average number of branches on the main stem at the height between 0.9 and 1.5 m and their basal diameters were of 8.1 and 14.3 mm, respectively, in ‘Manzanilla de Sevilla’ and of 6.8 and 14.6 mm in ‘Manzanilla Cacereña’. These values were markedly different to those of ‘Arbequina’, that showed the highest number of branches (14.8) and the lowest basal diameter (11.6 mm). Thus, branching efficiency in the hedgerows of both Manzanillas cultivars (around 0.5-0.6 No mm^-1^) was significantly lower than in ‘Arbequina’ (1.3 No mm^-1^). On the other hand, ‘Manzanilla de Sevilla’ produced an average of 78.4 shoots per branch (45.1-133.1) and ‘Manzanilla Cacereña’ 47.2 (40.3-54.4), and the shoots had similar mean values of length (≈14.5 cm), number of nodes (8.0), and basal diameter (2.3 mm). The highest differences were found in 2019. In comparison with ‘Arbequina’, this cultivar showed a lower number of shoots per branch (39.9), that had a similar length and number of nodes to the shoots of ‘Manzanilla de Sevilla’ and ‘Manzanilla Cacereña’, but were thinner, as indicated by the smaller basal diameter.

**Table 4:** Hedgerows structure for the studied cultivars and 'Arbequina' growing in super-high-density at the 10^th^, 11^th.^ and 12^th^ year after plantation.

Cultivar	Year	Branches^1^			Shoots^2^			
		Number	Basal diameter	Branching efficiency	Number	Length	Nodes	Basal diameter
			(mm)	(No mm^-1^)	(No Branch^-1^)	(cm)	(No)	(mm)
‘Manzanilla de Sevilla’	2017	8.4	13.0 ab	0.7 ab	45.1	16.0	8.9	2.4
	2018	9.0 ab	14.5	0.6 ab	57.1	9.6 a	7.1 a	2.0 a
	2019	7.0 a	15.2	0.5 a	133.1 b	19.2 b	8.0 b	2.5 b
	Mean	8.1 A	14.3 B	0.6 A	78.4 B	14.9	8.0	2.3 B
‘Manzanilla Cacereña’	2017	7.0	14.6 b	0.6 a	54.4	11.7	7.4	2.1
	2018	6.6 a	15.2	0.4 a	46.8	24.5 b	12.3 b	2.9 b
	2019	6.8 a	14.1	0.5 a	40.3 a	6.8 a	4.2 a	1.7 a
	Mean	6.8 A	14.6 B	0.5 A	47.2 AB	14.3	8.0	2.3 B
‘Arbequina’^3^	2017	13.0	9.8 a	1.4 b	25.7	9.3	7.2	1.9
	2018	13.4 b	13.5	1.0 b	28.0	9.2 a	6.9 a	1.7 a
	2019	18.0 b	11.7	1.6 b	65.8 ab	10.7 ab	6.3 ab	1.8 a
	Mean	14.8 B	11.6 A	1.3 B	39.9 A	9.7	6.8	1.8 A
Cultivar		****	**	****	**	ns	ns	***
Year		ns	ns	ns	**	ns	*	ns
Cultivar x Year		ns	ns	ns	*	***	***	***

^1^ Branches growing from the central leader at heights between 0.90 and 1.5 m.

^2^ Shoots on each of the three basal branches at the same heights.

^3^ Adjacent hedges of the same age, design and management, and average height of 2.6 m, width of 1.8 m and trunk diameter of 74.7 mm.

Different lowercase letters indicate significant differences between varieties for each year by Tukey test (P<0.05) in one-way ANOVA test. Different uppercase letters indicate significant differences between varieties by Tukey test (P<0.05). The significance of the last three lines corresponds to the multivariate analysis: ns, non-significant differences. *, **, *** and ****, significant differences at P < 0.05, 0.01, 0.001 and 0.0001.

Important interactions between cultivar and year were found for almost all parameters related to hedgerows structure (**Tables 3**, **4**), which means that growth responds strongly to the climatic conditions in each season.

## 4 Discussion

Results of this work show high fruit yield in the hedges of the two studied cultivars from the first year of study (**Figure 2**) despite the incidence of *Pseudomonas savastanoi* pv. *Savastanoi*. This bacterium causes olive tree tuberculosis, a disease characterized by the presence of galls that limit production. The spread of this disease in recent decades has been associated, in part, with branch injuries caused by mechanized harvesting systems (Trapero et al., 2025). In any case, results highlight the higher productivity and yield efficiency of ‘Manzanilla Cacereña’ hedgerows, that achieved exceptional cumulative yield by the end of the study, 65% higher than Manzanilla de Sevilla cultivar (**Figure 3**). The former practically doubled the efficiency in terms of production per m² of external surface area, that is related to the smaller size of its hedges, as they were shorter and narrower (**Table 3**). The preeminence of ‘Manzanilla Cacereña’ was evident from the first year of the study (with 17.7 t ha^-1^), that took place five years after planting. The precocity of first bearing reported in commercial orchards and breeding trials may be behind the higher yields of ‘this cultivar’.

The ‘Manzanilla de Sevilla’ hedgerows, although being less productive, showed fruits with higher commercial value because they were larger (in terms of weight and volume), more spherical and symmetrical compared to those of Manzanilla Cacereña cultivar (**Table 1**). Consequently, the ‘Manzanilla de Sevilla’ tended to produce larger commercial grades, while the ‘Manzanilla Cacereña’ fruits were usually more concentrated in medium and small grades (**Figure 2**), which is in agreement with the works of Morales-Sillero et al. (2014, 2023).

Fruit yield conditioned fruit size (**Figure 4**), although the correlation between both traits was weak (R²≈ 0.34), in agreement with Centeno et al. (2019). For ‘Manzanilla Cacereña’, the extremely high yield efficiency shifts the production towards smaller commercial grades compared to ‘Manzanilla the Sevilla’. However, alternate fruit bearing was observed in both cultivars (**Figure 3**). Thus, in high-yield years, such as 2017, the tree distributed its resources among more fruits, resulting in smaller sizes. Conversely, in low-yield years (such as 2018 for ‘Manzanilla de Sevilla’ or 2019 for ‘Manzanilla Cacereña’), the fruits were significantly larger (**Figures 1**, **2**). This pattern has been also reported for cultivars grown in super-high-density hedgerows destined to oil production (Centeno et al., 2019; Díez et al., 2016; Gómez-del-Campo et al., 2020; Pérez-Rodríguez et al., 2024). Alternate bearing is a complex phenomenon resulting from various factors. The main trigger is a high fruit load, which in a year of high yield restricts vegetative growth and affects fruit production in the following year of low yield. This leads to strong competition for carbohydrates and mineral nutrients. While it depends on the cultivar, the orchard’s environmental conditions and management (i.e., irrigation, pruning, and fertilization) can also influence this phenomenon (Connor et al., 2014). Thus, a higher alternate bearing index (0.56) was reported for ‘Manzanilla Cacereña’ in hedgerows grown in cold environments (Centeno et al., 2019). Regarding the lack of synchronization in the alternate bearing found in this work (**Figure 3**), the earliness of ‘Manzanilla Cacereña’ could also explain this pattern, although there is not data to demonstrate this hypothesis.

On the other hand, concerning fruit yield, it is worth noting the drop in production of ‘Manzanilla Cacereña’ hedgerows in 2019, approximately 35% lower than the previous year (**Figure 3**). This occurred after three years of high yields (from 15 t ha^-1^ in 2016 to 20 t ha^-1^ in 2017 and 2018). Furthermore, 2019 was drier than 2018, with a 47% decrease in rainfall and a 35% decrease in irrigation water. The application of N-P-K and Ca fertilizers was also lower, by approximately 30%. These results underline water availability as a key factor in ensuring the yield and fruit quality of table olive cultivars in super-high-density hedgerows. However, lower amounts of water below the approximately 300 mm of this study (on average) can be applied with irrigation scheduling based on tree water status and considering the phenological stage of the trees (i.e., with controlled deficit irrigation), provided that the water required during pit hardening is saved beforehand, and that an adequate rehydration is ensured in the weeks prior to harvest (Morales-Sillero et al., 2024; Sánchez-Piñero et al., 2024). This is crucial because environmental conditions in table olive producing areas tend to become increasingly challenging, with drier, hotter summers and warmer winters, which also leads to water scarcity for irrigation (Fraga et al., 2020).

Mechanical harvesting is also a key factor for the success of super-high-density hedgerows. The results of this work confirm the high efficiency of over-the-row harvesting in terms of fruit collection (≥96%, with less than 3.5% of fruit falling to the ground) and the time required to harvest, that was around two hours per hectare or less (**Table 2**), in agreement with Morales-Sillero et al. (2014). However, the quality of the olives after harvesting determines the economic success of this cropping system. There can be consequences like visual defects, loss of texture and problems during the fermentation process, all of which involve commercial rejection (Rallo et al., 2018). The greater damage of the olives after mechanical harvesting, both at the epicarp and mesocarp, is caused by the impact suffered by fruit falling through the canopy into the harvester and also by the handling and transportation to the factories (Pérez-Ruiz et al., 2018). Structural alteration of the mesocarp or pulp (including tissue rupture, and reduction in cell wall thickness) extends beyond the epicarp. The main visible symptom is tissue darkening due to cell rupture, the release of phenolic compounds (e.g., oleuropein derivatives) and subsequent oxidation reactions mediated by polyphenol oxidases (PPO). Fruit damage may not be visible in the field but may appear during transport or processing, depending on the cultivar, and it increases significantly between 4 and 24 hours after impact (Jiménez et al., 2016).

Specifically, ‘Manzanilla Cacereña’ stood up for its greater fruit physical integrity after manual and mechanical harvesting in every year of the studied period, with a lower bruising incidence and percentage of cut fruits (0% in 2018, despite a record production of 20 t ha^-1^) versus ‘Manzanilla de Sevilla’ fruits (≈100% of bruised fruits and up to 18% of cut fruits). Therefore, there is no doubt about the higher susceptibility of ‘Manzanilla de Sevilla’. Differences in varietal response have been related to the structure and composition of the cuticle (Domínguez et al., 2026; Jiménez et al., 2017). Both cultivars showed similar amount of cuticle per surface area, but ‘Manzanilla Cacereña’ fruits had a thicker cuticle and higher content of total waxes and phenols, which do not change after harvesting by the over-the-row harvester. In ‘Manzanilla de Sevilla’, total waxes decreased slightly but significantly after mechanical harvesting.

Previous studies done in the same orchard proved the importance of the environmental conditions at harvesting, as olive fruit damage was reduced when harvested at dawn (Morales-Sillero et al., 2023). Besides, the appropriate adjustment of parameters of the over-the row machine is necessary to maximize fruit removal while reducing the damage in fruits and the loss of biomass in trees (Pérez-Ruiz et al., 2018). Thus, the speed and beating frequency were set at 2 km h^-1^ and 430 Hz in the period 2015–2020 instead of 3 km h^-1^ and 470 Hz (2012, 2014) which were the conditions widely used in olive hedgerows for oil production until then. However, adjusting these parameters did not guarantee the absence of cuts in the fruit in any cultivar (**Table 2**), and this type of damage was highly related to fruit yield in the case of ‘Manzanilla Cacereña’ (**Figure 5**). Therefore, minimizing the damage caused by the harvester to the fruit, particularly cuts, remains a key challenge for hedgerow cultivation of ‘Manzanilla de Sevilla’ (as well as for any other cultivar susceptible to fruit damage), as it significantly affects subsequent processing in the industry and depreciates its market value. To date, postharvest fast immersion of mechanical harvested fruits in a diluted and cooled NaOH solution is recommended for ‘Manzanilla de Sevilla’ fruits intended for Spanish-style green olive elaboration whatever the type of machine harvester (Morales-Sillero et al., 2014; Castro-García et al., 2015; Zipori et al., 2021), in particular if the groves are located more than one hour away from the processing plants.

The analyzed growth parameters reveal that `Manzanilla de Sevilla' hedgerows were markedly more vigorous and porous than ‘Manzanilla Cacereña’ hedgerows, which exhibited a more restricted growth. In any case, for both cultivars, the architecture of the hedgerows was not as "compact" as for `Arbequina (**Table 3**)’, a low-vigor cultivar amply grown for oil production with high branching efficiency (Rosati et al., 2013), but they were adapted to mechanical harvesting (**Supplementary Figure 1**). Therefore, `Manzanilla de Sevilla' and `Manzanilla Cacereña' super-high-density hedgerows requires carefull growth control to promote light penetration and prevent the self-shading tendency in lower-canopy layers that is usually observed in this type of orchards with a negative impact on fruit yield and quality (Rosati et al., 2021; Trentacoste et al., 2021; Rallo et al., 2024). This should be achieved through proper pruning, irrigation, and fertilization management.

## 5 Conclusion

There is no doubt about the growing interest in the table olive sector for super-high-density cultivation, as it effectively addresses labor shortages by enabling full mechanization using over-the-row harvesters. Nonetheless, until well suited new bred cultivars and/or secondary local varieties with less vigor and resilient to environmental conditions, among other aspects, become available, the cultivation of traditional table olive cultivars such as the two here studied is the current alternative. The Manzanilla Cacereña cultivar is better suited for this system, standing out for its high productivity, yield efficiency, and superior resistance to mechanical damage, showing significantly lower incidence of bruising and cuts. In contrast, while ‘Manzanilla de Sevilla’ produces larger, high-value fruits, its extreme susceptibility to browning during mechanical harvesting requires immediate post-harvest treatments, such as immersion in a diluted and cooled NaOH solution, to prevent commercial rejection. Consequently, the long-term success of this cultivation system depends on precise agronomic management, including strategic pruning to control vegetative vigor and ensure adequate light penetration, as well as careful irrigation, fertilization, and harvest management to maintain consistent yields and high fruit quality.

## Data Availability

The datasets supporting the findings of this study are publicly available at https://doi.org/10.12795/11441/186640. Further Inquiries should be directed to the corresponding author.
